# Perinatal mental disorders and suicidal risk among adolescent mothers living in urban areas of Cameroon

**DOI:** 10.3389/fpsyt.2024.1306440

**Published:** 2024-06-11

**Authors:** Joël Djatche Miafo, Daniel Nzebou, Beat Stoll, Joris Cathel Yimga Ngambia, Saskia von Overbeck Ottino, Amir Moayedoddin

**Affiliations:** ^1^Research Department, Uni-Psy et Bien-Être (UNIPSY), Yaoundé, Cameroon; ^2^Actions en Santé Publique (ASP), Geneva, Switzerland; ^3^Institut de Santé Globale, Université de Genève, Geneva, Switzerland; ^4^Service de psychiatrie de l’enfant et de l’adolescent, Hôpitaux Universitaires de Genève (HUG), Geneva, Switzerland

**Keywords:** perinatal mental health, perinatal period, perinatal mental disorders, adolescents mothers, suicidal risk, unsatisfied social needs, Cameroon

## Abstract

**Background:**

In sub-Saharan Africa the birth rate among teenage mothers is the highest in the world. In 2021, there would be 6,114,000 births for 15–19-year-olds in this part of the world. In Cameroon, the fertility rate among adolescents aged 15–19 is 24%. However, there is a significant lack of data on the mental health of teenage mothers. Given the biopsychosocial conditions of the perinatal period and adolescence, we hypothesise that the prevalence of mental disorders and the risk of suicide is very high in Cameroon. The aim is therefore to determine the prevalence of perinatal mental disorders and suicide risk among adolescent mothers in urban areas of Cameroon.

**Methods:**

Following ethical approval of the submitted protocol, we recruited adolescent mothers and data were collected using diagnostic interviews based on the DSM-5, PDM-2 and MINI guidelines. The types of sampling used were typical and incidental. Data were tabulated with Epidata 3.1 and processed with SPSS 25.

**Results:**

66.4% of adolescent mothers were diagnosed with a mental disorder and 27.4% with suicidal risk. It was found that there was a link between mental disorders and suicidal risk (p<0.001), with mothers at suicidal risk having an 8.4 times greater risk of having a mental disorder (OR=8.423). Linear regression confirmed the statistically significant relationship between perinatal mental disorders and suicidal risk. 31.1% of the total variance in suicidal risk was explained by mental disorders. The regression coefficients for mental disorders with a p<0.05 value is: perinatal depression (-0.279), post-partum psychosis (-0.133), trauma disorder (-0.034), generalised anxiety disorder (-0.008) and conduct disorder (-0.020).

**Conclusions:**

Our hypothesis is confirmed, because the prevalence of 66.4% of mental disorders and 27.4% of suicidal risk are significantly high in Cameroon. In some way, the disorders predict suicidal risk, because the less an adolescent mother has one of these pathologies during the perinatal period, the less she will be at risk of suicide. More research of this kind is needed to contribute in providing more data, including solutions to address the morbidity and mortality problems associated with the mental health of teenage mothers.

## Introduction

The possibility of becoming pregnant and having a baby is a reality for adolescent girls around the world. This reality is determined by conditions of biological, psychological and social vulnerability. The perinatal period runs from the first day of pregnancy to the end of the baby’s first year of life (1–3). The mental disorders, states of psychological distress or perinatal mental problems, studied by the perinatal psychiatry, have a high prevalence and are recognised as major contributors to maternal morbidity and mortality in adolescent girls, and to health and development risks for babies (4, 5). Perinatal psychiatry is a discipline that lies at the interface between adult and child psychiatry. Its aim is to study the mental disorders that occur in one or other of the parents during the perinatal period, as well as the specificities of the development of the foetus, the newborn and the infant in this context. The disorders that appear during this perinatal period and that are dependent on it are called perinatal psychiatric disorders (6). According to the theory of human birth, mental illness can be understood as a pathology of the human relationship immediately after birth and during the first year of life (7). In high-income countries, the prevalence of maternal depression in this population group is 48% (3). In low- and middle-income regions such as sub-Saharan Africa, the perinatal mental health of adolescent mothers is neglected and, in some areas, virtually non-existent. However, some research has been carried out, although not in all areas (8). Yet in sub-Saharan Africa, the birth rate among teenage mothers is the highest in the world. In 2021, there would be 6,114,000 births by 15–19-year-olds in this part of the world (9). However, studies on the prevalence of perinatal mental disorders in sub-Saharan Africa have focused solely on perinatal depression or its symptoms. Prevalence rates ranged from 10.1% to 94% (8). With regard to the problem of suicide in this segment of the population, only two qualitative studies have been carried out in Kenya and South Africa on the factors associated with suicidal behaviour (10). In Central Africa, particularly Cameroon, there is one study on the prevalence of symptoms of perinatal depression (1). In this country, the fertility rate among adolescents aged 15–19 is 24% (11). It therefore seems crucial for us to seek information on the prevalence of perinatal mental disorders and psychological distress in this category of the population. Our hypothesis is that the prevalence of perinatal mental disorders is very high and that these can predict the occurrence of suicidal risk. The aim of our study is to determine the prevalence of perinatal mental disorders and suicidal risk, and the link between the two, in adolescent mothers during the perinatal period, in urban areas in Cameroon. Beforehand, their socio-demographic characteristics and unmet social needs will be identified.

## Methods

Determining the prevalence of perinatal mental disorders and suicidal risk, including the relationship between the two, requires a scientifically proven approach.

### Ethical considerations

The study procedures were carried out in accordance with the ethical principles and obligations to which researchers are bound by the Declaration of Helsinki (12). All participants were informed of the study and provided written consent. They were informed that if they wished, they could withdraw from the study at any time. Ethical approval was granted by the National Ethics Committee for Human Health Research (CNERHH) in Yaoundé, after evaluation of our research protocol. The approval number is: 2014/03/436/L/CNERSH/SP.

### Framework of the study

This study is the research dimension of an action research project, which combines, in a single movement, a research rationale to produce knowledge and an action rationale to act, modify and improve a situation or an activity (13). In Cameroon, the partners involved were: the Ministry of Public Health (MINSANTE), the Ministry for the Promotion of Women and the Family (MINPROFF), Le Réseaux National des Associations de Tantines (RENATA), Uni-Psy et Bien-Être (UniPsy) an organisation of mental health professionals. And in Geneva, the University of Geneva, Action en Santé Publique (ASP) a non-governmental organisation, the Department of Child and Adolescent Psychiatry (SPEA) of the Geneva University Hospitals and the Worl Health Organisation (WHO). Skills were enhanced by training a team of 71 front-line workers (nurses, social workers, psychology students, junior psychologists). These skills included the use of the World Health Organisation’s (WHO) Mental Health Global Action Plan (mhGAP) based on the Diagnostic and Statistical Manual of Mental Disorders (14, 15), the Edinburgh Postnatal Depression Scale (EPDS) (16), the Mini International Neuropsychiatric Interview (MINI) (17), and other data collection instruments (questionnaires, interview guide, risk factor inventory). Supervision was provided by 8 2nd line clinical psychologists and 3 3rd line psychiatrists. The front-line workers administered the data collection tools, and the clinical psychologists and supervising psychiatrists made the diagnoses.

### Population and sample

For the purposes of this study, we will refer to all teenage mothers who are pregnant or have a baby aged one year or less, as teenage mothers. Our study population consisted of 1633 adolescent mothers in the perinatal period. Their place of residence was Yaoundé and its environs. With regard to their perinatal status, 827 were prenatal and 783 postnatal. Recruitment was continuous for 4 years 8 months, from April 2014 to December 2018. Inclusion criteria were being aged 20 years or less, pregnant or having a child no older than 12 months. The exclusion criteria were being over 20 years of age, having a child over 12 months of age, not having fully completed the collection tools and not having been clinically assessed. The non-probability sampling technique was chosen. 2 types of sampling were carried out. Typical sampling was used for participants recruited at the hospital and through the personal network of carers, and accidental sampling for those recruited door-to-door (18).

### Procedure, tools and data collection

The research protocol was drafted, corrected and submitted for ethical approval. A framework for face-to-face and remote discussion was created for the action research project to facilitate the exchange of information and the coordination of the project. Training sessions lasting between three and five days were organised each year for the data collection team. The aim was to teach them how and when to conduct the interview, and to go through the EPDS, including the questionnaire and interview guide. The data was collected by means of directive and semi-directive interviews based on the sub-themes presented in **Table 1** below:

**Table 1:** Sub-themes addressed during data collection.

Sub-themes	Contents
Socio-demographic profile	Age, occupation, perinatal status, marital status, religion, sibling rank, level of education, region of origin, living area (whether close to home or not), contacts
Evaluation	Context of the meeting, social needs, perceptions of mental health, baby’s psychological and physical state, baby’s diet
Perinatal and parental experiences	State of relationship with partner, state of relationship and social interactions with others during the perinatal period, pre-, per- and postnatal experience, changes and evolution of the state of health of the mother and child, transformations linked to parenthood and the quality of the mother-baby relationship.
Psychopathological aspects and mental disorders	Screening and diagnostic tools: Edinburgh Postnatal Depression Scale (EPDS), Mini International Psychiatric Interview (MINI) for suicidal risk, DSM-5 for diagnosing mental disorders, PDM for diagnosing complex psychotrauma and mother-baby relationship disorders, presence or absence of a dysfunctional family (7 open questions are asked for this aspect and the themes are as follows: communication, roles, conflict resolution, material satisfaction, emotional reactions, emotional involvement, behavioural control).
Risk factors for perinatal mental disorders	5 categories: circumstances of pregnancy and motherhood, health issues, social risk factors, negative experience of childbearing or childbirth, state of marital and family relationships.

### Collection tools

The diagnosis of a mental disorder is made by psychiatrists, psychologists or other care providers trained and authorised to do so. It is made using a number of methods based on questionnaires or observations, which are in turn based on tried and tested and scientifically validated theoretical and clinical corpus. This is the case for perinatal and adolescent mental disorders. When it comes to diagnosing adolescents, given the period of development, a certain number of psychopathological symptoms may be observed, when in fact they are not. The diagnosis must take account of both diachronic and synchronic data. This is why, in addition to the presence of signs and symptoms, the diagnosis will take into account parameters such as: the individual’s history, environmental circumstances, the degree of rupture with past functioning, family interactions, disturbance of habitual psychic functioning, maladjustment to external reality, biopsychosocial risk factors, the intensity and permanence of signs and symptoms (19). The aim of taking these parameters into account is to increase the reliability and viability of the diagnostic criteria for teenage mothers. We did not find any specific scales for screening and diagnosing perinatal mental disorders in adolescent mothers. The above comments have been taken into account in our work, as can be seen from the above-mentioned evaluation sub-themes (socio-demographic profile, evaluation, perinatal and parental experiences, psychopathological aspects and mental disorders, risk factors for perinatal mental disorders).

The three standards used to diagnose mental disorders are: the Diagnostic and Statistical Manual of Mental Disorders 5th edition (DSM-5), the Mini International Neuropsychiatric Interview (MINI) and the Psychodynamic Diagnostic Manual (PDM). The former provides a clear and systematic description and classification of mental disorders (15). It is intended for mental health professionals and is an international reference. Clinical psychologists and psychiatrists have used this reference to confirm the diagnosis of perinatal mental disorders. The second standard, the MINI, is a short clinical interview structured interview that allows researchers to diagnose psychiatric disorders according to the DSM-IV. The interview was designed for epidemiological studies and multicentre clinical trials (17). When used correctly, it can also be used to make a diagnosis. It measures the absence or presence of an illness, based on rigorous criteria that correspond to those of the Diagnostic and Statistical Manual of Mental Disorders. In this study, we used the French version 5.0.0 only to assess suicidal risk, which is either non-existent, mild, moderate or high. The third reference, the PDM, is the result of an effort to formulate a psychodynamically oriented diagnosis that bridges the gap between clinical complexity and the need for empirical and methodological validity (20, 21). One of the aims of this manual is to complement the DSM and the International Classification of Diseases (ICD). In the context of our research, we used it to make diagnoses of complex psychotrauma and mother-baby relationship disorders (22). All these diagnostic criteria are also those described by the ICD 10: bipolar disorder, depressive episodes with somatic syndrome, neurotic disorders, obsessive-compulsive disorder, reaction to stress factors (adjustment disorder, post-traumatic stress disorder, etc.). Symptoms may have mood-related aspects such as sadness, guilt, self-blame, negative thoughts, etc. Others may express anxiety: irritability, sleep disturbance, panic, etc. Occasionally, mothers may exhibit psychotic signs described in the ICD 10, such as delusions, hallucinations, etc. (23). To sum up, in the light of the above, this approach is a strength, since it relies on several complementary elements to reach a diagnosis. On the other hand, the diagnosis may be limited by the fact that the mothers are still in their teens. The health workers who collected the data did not participate in either the tabulation or the analysis of the data.

### Data analysis

The data were analysed using Epidata 3.1 and processed using SPSS 25. Descriptive statistics were first performed. This consisted in highlighting the different frequencies of the modalities of our variables. Next, we calculated the chi-square (X²), the odds ratio (OR) and the relative risks (RR) for the cross-tabulations. Finally, we performed a linear regression to determine the predictive effect of mental pathologies on suicidal risk factors among teenage mothers.

## Results

The general objective was to determine the prevalence of perinatal mental disorders and suicidal risk among adolescent mothers during the perinatal period in Cameroon. To achieve this, we present the sociodemographic characteristics and unmet social needs. We will then use descriptive statistics for prevalence, after collecting data using the DSM-5, PDM and MINI tools. Finally, we will analyse the link and relationship between the different variables using inferential statistics.

### Socio-demographic characteristics of teenage mothers

1633 teenage mothers took part in the study. As shown in the **Table 2** 51.4% were pregnant (prenatal) and 48.5% had a child up to one year old (postnatal). The mean age was 17.91 years and the oldest age group was the age group represented by 18–20 years, i.e., 68.4% of participants. 75% of them were single mothers. In terms of level of education, lower secondary education was the most represented, with 47.8% included. In terms of gestational age, most were between the second and third trimesters, 23.5% and 19.5% respectively. 38% had a single child and 7.8% had 2 children. The average age of the babies was 5.31 months. The majority of these mothers (46.8%) were recruited in health facilities, while 39.1% were recruited door-to-door. When asked what their unmet needs were, 58.7% said money, 55.6% moral support from family and friends, and 47.3% knowledge of how to look after a pregnancy or a baby. Given these characteristics and unmet social needs, one may ask what the prevalence of suicidal risk and perinatal mental disorders is in this population.

**Table 2:** Socio-demographic characteristics of teenage mothers.

Characteristics	Frequency	n% (IC=95%)
Perinatal state		
Prenatal	840	51.4 (48.8–53.8)
Postnatal	793	48.5 (46 -50.)
Age (M=17.91; Min=12; Max=20; SD=1.30)		
< 15 years	84	5.1 (4.2–6.4)
15 - 17 years	416	25.5 (23.6–27.9)
18 - 20 years	1117	68.4 (66.8–71.3)
Marital status		
Single	1224	75.0 (73.3–78)
In a cohabiting relationship	274	16.8 (15.2–18.9)
Married	113	6.9 (5.8–8.4
Divorced	1	0.1 (0–0.3)
Widow	1	0.1 (0–0.3)
Level of education		
Out of school	35	2.1 (1.6–3.1)
Primary education	171	10.5 (9.4–12.5)
Lower secondary education	780	47.8 (47.2–52.2)
Upper secondary education	528	32.3 (31.3–36.1)
University level	55	3.4 (2.7–4.5)
Gestational age (M=5.72; Min=1; Max=9; SD=1.98)		
1 - 3 month	126	7.7 (5.7–8.2)
4 - 6 month	384	23.5 (21.4–25.6)
7 - 9 month	318	19.5 (17.6–21.4)
Age of child (M=5.31; Min=1; Max=12; SD=3.54)		
1 - 3 month	301	18.4 (16.5–20.3)
4 - 6 month	200	12.2 (10.6–13.8)
7 - 9 month	132	8.1 (6.7–9.4)
10 - 12 month	127	7.8 (6.4–9.1)
Number of children (M=1.21; Min=1; Max=3; SD=0.45)		
1	621	38.0 (35.6–40.4)
2	128	7.8 (6.4–9.1)
3	15	0.9 (0.4–1.3)
Meeting the teenage mother		
Health Facilities	764	46.8 (44.8–49.7)
Door to door	639	39.1 (37.1–42)
Health worker network	213	13.0 (11.6–14.9)
Unsatisfied social needs		
Money	959	58.7 (56.3–61.1)
Moral support	908	55.6 (53.2–58.0)
Practical support	602	36.9 (34.6–39.2)
Medical care for baby	138	8.5 (7.1–9.8)
Medical care for teenage mothers	163	9.9 (8.4–11.3)
Knowledge about maternity	773	47.3 (44.9–49.7)
Legal assistance	32	2.0 (1.3–2.7)
Training/guidance	477	29.2 (27.0–31.4)
Shelter	48	2.9 (2.1–3.7)
Employment	350	21.4 (19.4–23.4)
School Enrolment	302	18.5 (16.6–20.4)

### Prevalence of suicidal risk and perinatal mental disorders among teenage mothers

Determining the prevalence of suicidal risk and perinatal mental disorders is one of the specific aims of our research. As indicated above, we administered interviews based on the DSM-5, PDM and MINI tools to make diagnoses. 1085/1633 teenage mothers were diagnosed as suffering from at least one mental pathology, as shown in **Table 3**, a rate of 66.4%. Broken down, the rate was 39.9% for prenatal teenage mothers and 32.6% for postnatal teenage mothers, relative to the total sample. The prevalence of maternal depression was 60.9% (994/1633). 176 suffered from perinatal anxiety (generalised anxiety), a prevalence of 10.7%. With regard to mother-baby relationship disorder, 6.1% were diagnosed as positive. Trauma disorder was also observed, with a prevalence of 7.8% (128/1633). It is distinguished by its two dimensions: post-traumatic stress disorder, 4.9%, and complex traumatic disorder, 2.9%. 4.1% had a behavioural disorder. 1.1% were addicted to substances (alcohol, tobacco and drugs). Among teenage mothers living in dysfunctional families, 411, or 25.2%, were in this category. It was found that 27.4% of teenage mothers were at mild risk of suicide, while 2.7% were at medium risk and 7.3% at high risk.

**Table 3:** Prevalence of mental disorders and suicidal risk among teenage mothers in the perinatal period in Cameroon.

Variables		Frequency	n % (IC 95%)
Teenage mothers with at least one mental disorder		1085	66.4 (64.1–68.7)
Prenatal teenage mother		545	33.9 (31.6–36.1)
Postnatal teenage mother		525	32.6 (30.3–34.8)
Depression		994	60.9 (58.5–63.2)
Psychosis		12	0.7 (0.4–1.3)
Traumatic disorder		128	7.8 (6.7–9.3)
Post-traumatic stress disorder		80	4.9 (4.6–5.1)
Complex traumatic disorder		48	2.9 (2.6–3.1)
Substance use disorder		18	1.1 (0.7–1.7)
Generalised Anxiety Disorder		176	10.7 (9.3–12.4)
Disturbance in the mother-baby relationship		101	6.1 (5.1–7.5)
Conduct disorder		67	4.1 (3.2–5.2)
Suicidal risk	Mild	448	27.4 (25.3–29.7)
	Medium	44	2.7 (2–3.6)
	High	120	7.3 (6.1–8.7)
Dysfunctional family		411	25.2 (23.1–27.3)

### Unsatisfied social needs, mental disorders and suicide risk

The association between subjects having had at least one mental disorder and those having at least one need was significant (p≤0.001), as was the case for those at risk of suicide and having at least one unmet need (p≤0.001). In addition, as presented in **Table 4**, teenage mothers with at least one unmet social need were 0.17 times more likely to have a mental pathology and 0.28 times more likely to be at risk of suicide during the perinatal period.

**Table 4:** Correlation between unmet needs, mental disorders and suicide risk.

		Adolescent mother with at least one need		*P-value*	OR
		Yes, n (%; IC)	No, n (%; IC)	<0.001	0.177
**Teenage mothers with at least one mental disorder**	Yes	1054 (64.5;62.1–66.8)	31 (1.9; 1.2–5.5)		
	No	470 (28.8;26.6–30.9)	78 (4.8;3.7–5.8)		
		**Teenage mother with at least one need**		<0.001	0.289
**Teenage mothers at risk of suicide**	Yes	595 (36.4;34–38.7)	17 (1.0;0.5–1.4)		
	No	929 (56.9;54.4–59.3)	92 (5.6;4.4–6.7)		

In addition, we want to verify the confounding effect or effect modification between having at least one need, in association with having at least one pathology and suicidal risk. Indeed, we find that having at least one pathology is an effect modifier (OR1 = 2.223≠ OR2 = 1.283) of the association between having at least one need and having a suicidal risk. Furthermore, we have a quantitative type interaction, because the relationship between having a suicidal risk and having at least one need, go in the same direction, i.e. increasing. It is stronger in subjects with at least one pathology than in those with no pathologies.

### Link between mental disorders and suicidal risk

The link between suicidal risk and mental disorders was significant (p≤0.001), as shown in **Table 5**. In addition, teenage mothers at risk of suicide were 8.4 times more likely to have a mental illness.

**Table 5:** Correlation between suicide risk and mental disorders.

	Teenage mothers with at least one mental disorder			
**Suicidal Risk**	Yes) n (/%; IC)	No) n (/%; IC)	*P-value*	OR (RR)
	552 (33.8;31.5–36.2)	60 (3.7;2.8–4.7)	<0.001	8.423

Furthermore, the F statistic of 122.536 with a p-value of <0.001 shows that there is a statistically significant relationship between the various mental disorders that have a p-value of less than 0.05 and the fact of haven presented a suicidal risk. This is why these mental disorders explain 31.1% of the total variance in cases of suicidal risk among adolescent mothers. In fact, as presented in **Table 6**, the regression coefficients for mental disorders with a p<0.05 value are: depression (-0.279), post-partum psychosis (-0.133), trauma disorders (-0.034), generalised anxiety disorder (-0.008) and conduct disorder (-0.020). This implies that, on the whole, these mental disorders have negative and significant effects on the presence of suicidal risk observed in adolescent mothers. In other words, the less an adolescent mother suffers from one of these mental disorders during the perinatal period, the less likely she is to commit suicide.

**Table 6:** Linear regression of suicidal risk and mental disorders.

Summary of models										
Model		R	R-two		Adjusted R-two	Standard error of estimate				
1		.558^a^	.311		.309	.403				
ANOVA^a^										
Model				Sum of squares		ddl		Mean square	F	Sig.
1	Regression			119.144		6		19.857	122.536	.000^b^
	of Student			263.497		1626		.162		
	Total			382.641		1632				
Coefficients^a^										
Model				Model Non-standardised coefficients			Standardised coefficients		t	Sig.
				B		Standard error	Beta			
(Constante)				1.899		.016			116.905	.000
Depression				-.279		.022	-.281		-12.877	.000
Postpartum Psychosis				-.133		.059	-.047		-2.267	.023
Traumatic disorder				-.034		.008	-.094		-4.434	.000
Generalised anxiety disorder				-.008		.004	-.047		-2.251	.024
Conduct disorder				-.020		.005	-.089		-4.292	.000

a=dependent variable (subjects with at least one suicidal ideation); b=predictors or constancy, i.e. the set of variables retained in the model, i.e. depression, postpartum psychosis, traumatic disorder, general anxiety.

## Discussion

The aim of our study was to determine the prevalence of perinatal mental disorders and suicidal risk among adolescent mothers in Cameroon, and the correlations between these two phenomena. To do this, we identified the field, defined a framework, a sample and collection procedures using the DSM, PMD and MINI tools. A quantitative, descriptive and inferential analysis of the data was carried out and the results obtained.

Our results indicate that 66.4% of teenage mothers in urban areas of Cameroon suffer from at least one mental disorder. This is equivalent to almost 2/3 of teenage mothers in need of treatment. This 66.4% prevalence rate of mental disorders in Cameroon is close to the 72.2% rate of mental health needs identified among adolescent mothers in sub-Saharan Africa from 2013 to 2021 (24). In comparison, our study goes beyond mental health problems and identifies mental disorders using diagnostic instruments. In addition, ours is more precise and focuses on teenage mothers (aged 11–20), whereas in this research, the study populations go beyond the category of teenage mothers and include young mothers (aged 20–24), parents and certain health professionals. The added value of our results is that they go beyond simply presenting the prevalence rate of perinatal depression and the symptoms of perinatal depression in adolescent mothers, as done by other studies in sub-Saharan Africa (Cameroon, Zimbabwe, Nigeria, Ghana, Ethiopia, Tanzania, Uganda, Kenya, Malawi, South Africa) (8). The prevalence of traumatic disorders, generalised anxiety disorders, mother-baby relationship disorders, conduct disorders, substance-related disorders and postpartum psychosis were all highlighted in our research.

With regard to the prevalence of perinatal depression among adolescent mothers in Cameroon, our results show that it is 60.9%. In so-called developed countries, the rate is 48% (3). In sub-Saharan Africa, the rate varies between 8.8% and 94%. The higher rates can be explained by the fact that the populations were exclusively either rape survivors (25) or mothers of premature babies (26). From this point of view, the fact that our study population is more heterogeneous, the rate of 60.9%, seems to us to be closer to the reality of all adolescent mothers. In Central Africa, and Cameroon in particular, an initial study was carried out on the prevalence of depressive symptoms among teenage mothers. The prevalence was 70% (1). Other studies were carried out in Cameroon on a population of adult mothers, and the prevalence of postnatal depressive symptoms was 31.8% and 23.4% (27, 28). The new contribution of our research is that it went further by showing not only the prevalence of diagnosed perinatal depression, but also that of other mental disorders. Our result of 66.4% prevalence of mental disorders in adolescent mothers in urban areas of Cameroon is close to 68.9%, which is the prevalence of mental disorders in a Cameroonian population of children, adolescents and young people in the context of armed conflict and displacement/migration (29).

In our work, we found that having an unmet social need had a statistically significant effect on the presence of suicidal risk (p<0.001), including mental disorder (p<0.001). These results confirm the general trend that the presence of a mental disorder is associated with psychosocial factors (3, 8, 30). In fact, the risk of having a mental illness or being at risk of suicide was slightly higher among teenage mothers with at least one unmet social need (0.28 times and 0.17 times higher).

Our results show a prevalence rate of mild, medium and high suicidal risk of 27.4%, 2.7% and 7.3% respectively. To the best of our knowledge, this is the first study in sub-Saharan Africa to address the phenomenon in terms of the prevalence of suicidal risk among teenage mothers with a significant population (1633). A study carried out in Kenya on suicide instead used a qualitative approach to deal with this suffering among teenage mothers. The aim was to identify themes linked to the risk of suicidal behaviour (9). Another qualitative study in South Africa explored the link between pregnancy, HIV, violence and mental health problems, including suicidal ideation. The population of South Africa’s study consisted of pregnant mothers aged 15–24 years (31). We wanted to propose an attempt to explain the presence of suicidal risk. To do this, as described in the results section, we performed multivariate linear regression. The aim was to determine the independent predictors of suicidal risk on the basis of the mental disorders that were significantly associated with suicidal risk in the univariate analyses. This approach differs from those used in the Kenyan and South African studies, which are more qualitative (8, 10, 24, 30). Ours was carried out on a population 6 times larger, with a focus on a single phenomenon, namely mental disorders, in a quantitative and therefore generalisable approach. This is something new in sub-Saharan Africa. In the multivariate linear regression, after various iterations, individual predictors using standardised beta scores were examined. Thus, suffering from depression, psychosis, traumatic disorder, generalised anxiety disorder and conduct disorder explained the greatest variance (31.1%) in the relative burden of suicidal risk among pregnant and breastfeeding adolescents. Adolescent mothers at risk of suicide were 8.4 times (OR=8.423) more likely to suffer from a mental disorder.

Recent research suggests that it is imperative to develop personal identity before sexual orientation and possibly parenthood. Babies and children of teenage mothers are at greater risk of prematurity and low birth weight, followed by developmental delays and behavioural problems. There is also the theory of human birth, which posits that mental illness is a consequence of the disruption of the human relationship immediately after birth during the first year of life (5, 7). On the basis of these postulates and observations, the high prevalence rate of 66.4% of psychological distress among teenage mothers is understandable, on the one hand, and would suggest a significant future increase in mental illness, somatic disease and developmental delays in the Cameroonian population, on the other. The significant presence of risk factors and unmet social needs could also explain the high rate of perinatal mental disorders and suicidal risks among teenage mothers in Cameroon. A further study would shed more light on this hypothesis.

The multifactorial model could also explain this high rate of perinatal mental disorders. These include the young woman’s personality, her family history, her personal psychiatric and obstetric history, hormonal changes, the stress of childbirth and the importance of the family and social network (32–34). In view of the difficult histories, the high number of unmet social needs, and the biology of adolescent mothers who are still growing, we can understand the high prevalence of mental disorders (66.4%.) and suicidal risk (27.4%) in the Cameroonian context. Furthermore, in this perspective, another study (1) that explored risk factors among teenage mothers in Cameroon identified factors linked to depressive symptoms. These were: unwanted or planned pregnancy, being alone or separated, depression or anxiety during pregnancy, experience of abortion, domestic violence. Thus, although epidemiology in psychiatry and health developed somewhat late, we now have the possibility of determining the frequency of mental disorders (descriptive epidemiology) and identifying risk or vulnerability factors (analytical epidemiology). The same applies to perinatal psychiatry (33). It has therefore been possible to apply it in Cameroon to adolescent and adult mothers (1, 35).

In short, we believe that it is important in sub-Saharan and central Africa, and in Cameroon in particular, to know the prevalence of mental disorders and the risk of suicide among teenage mothers. Given that they account for a quarter of adolescent girls in Cameroon, it makes sense to know how widespread these mental disorders are among this population. From a public health planning and surveillance perspective, the prevalence of mental disorders among teenage mothers gives us an idea of the extent of the burden of mental disorders among them (36). The absence of this information in our context (Cameroon) prior to our study proved to be a major shortcoming, given the rate of 66.4%. There are a number of prevention and management solutions available. They involve developing comprehensive, quality reproductive and adolescent health services in schools and health facilities (37).

### Limitations and strengths

The first limitation of this study is that the population of teenage mothers was concentrated in an urban area. Teenage mothers living in rural areas and conflict zones in Cameroon were not taken into account. Another weakness is that the latest data collected dates from the end of 2018. In addition, the MINI could also have been used to establish other diagnoses, in addition to that of suicidal risk.

One of the strengths of this research is the method used to recruit teenage mothers, in particular door-to-door canvassing in the community. This approach enabled us to recruit mothers who were unable to visit the health facilities. This seems to us to have contributed to the representativeness of the sample. The large sample of 1633 also seems to us to be a strength.

## Conclusion

The prevalence of mental disorders among teenage mothers living in urban areas in Cameroon is 66.4%. The prevalence of suicidal risk is 27.4%. Our hypothesis about the high rate of these phenomena is confirmed. Ultimately, the paucity of research on mental disorders and suicidal risk among adolescent mothers (both quantitative and qualitative), in sub-Saharan, central Africa and Cameroon, constitutes a critical evidence gap. This gap limits evidence-based policy and programmatic responses, as well as national and regional development opportunities, to the negative consequences of perinatal mental disorders. Through this research, we have modestly attempted to contribute to filling these gaps. This research should be multiplied and solutions to combat the mental health problems of teenage mothers developed. And so, a few hypothetical solutions could be proposed, such as monitoring teenagers in schools, more sex education, more sexual information and public health aid centres, psychotherapy for teenagers at school, individual and group psychotherapy for young mothers. And finally, this work suggests future research in Cameroon and Central Africa. It would be interesting to determine the prevalence of disorders among adult mothers in several centres, to contextualise and validate screening and diagnostic tools for perinatal mental disorders, and to identify the most effective treatment approaches for perinatal mental disorders.

## Data availability statement

The original contributions presented in the study are included in the article/supplementary material. Further inquiries can be directed to the corresponding author.

## Ethics statement

The studies involving humans were approved by National Ethics Committee for Human Health Research (CNERHH) in Yaoundé. The approval number is: 2014/03/436/L/CNERSH/SP. The studies were conducted in accordance with the local legislation and institutional requirements. Written informed consent for participation in this study was provided by the participants’ legal guardians/next of kin.

## Author contributions

JD: Writing – review & editing, Writing – original draft, Visualization, Validation, Software, Resources, Project administration, Methodology, Investigation, Data curation, Conceptualization. DN: Writing – review & editing, Validation, Supervision, Methodology, Investigation, Formal analysis, Conceptualization. BS: Writing – review & editing, Visualization, Project administration, Methodology, Conceptualization. JY: Writing – review & editing, Methodology, Investigation. SV: Writing – review & editing, Visualization, Validation, Methodology, Formal analysis. AM: Writing – review & editing, Writing – original draft, Visualization, Validation, Project administration, Methodology, Conceptualization.
